# Investigation of Shear Strength and Microstructure Formation of Joined Ni Superalloys Using Ni Nanopastes

**DOI:** 10.3390/nano12183204

**Published:** 2022-09-15

**Authors:** Benjamin Sattler, Susann Hausner, Guntram Wagner

**Affiliations:** Group of Composites and Material Compounds, Chemnitz University of Technology, 09125 Chemnitz, Germany

**Keywords:** joining, nanojoining, nanopaste, Ni nanoparticle, alternative to brazing, nickel base superalloy, Alloy 247 DS, Inconel 718, additive manufacturing

## Abstract

By using Ni nanoparticles, the bonding of Ni base superalloys can be achieved with shear strengths well above 200 MPa in a joining process at comparatively low temperatures between 675 °C and 975 °C. This is enabled due to the high surface-to-volume ratio of nanoparticles, which leads to distinctly lower melting and sintering temperatures than those of the corresponding bulk material. The nanoparticles in this study are employed in high-metal nanopastes, whereby different chemical compositions of the pastes and different sizes of Ni nanoparticles were investigated. The results for the joining of Ni base superalloys showed that both size and composition had a significant influence on the achievable strengths. In addition, an extensive examination was conducted to reveal the influence of the process parameters joining temperature, holding time and joining pressure on the shear strengths as well as microstructure. It was shown that the temperature exerted the most influence on the strengths and the microstructure. The joining pressure also had a significant influence. The holding time, on the other hand, did not have a major influence on the strengths and in some cases even showed an unexpected behavior, as the values decreased for some combinations with longer holding time.

## 1. Introduction

In applications such as gas turbines, industrial furnaces or wherever high temperatures are present, nickel-base superalloys are widely used due to their excellent properties to withstand high-temperature and corrosive environments under mechanical stress [1,2,3]. When components made of these alloys must be assembled or repaired, various joining techniques, such as welding or brazing, are available. However, some restrictions must be considered for each of them. When welded for example, the properties of these alloys can suffer from segregation issues during solidification due to the material high diffusion rates [4]. Even if no melting occurs during a joining process as with brazing, recrystallization effects above 1100 °C negatively affect the structure of some single-crystal Ni base materials [5]. Moreover, the braze filler itself limit the maximum operating temperature of the brazed components and the containing melting point depression elements can lead to brittleness when intermetallic compounds are formed at the joint centerline [6,7].

In the course of the further development of the joining processes, a relatively new method, so-called nanojoining, has emerged as an alternative which take advantage of the remarkable properties of nanoparticles [8,9,10,11,12]. One of the main advantages is the possibility of low joining temperatures, since melting and sintering temperatures of nanoparticles are significantly lower than those of the corresponding bulk material due to their high specific surface area or high surface-to-volume ratio, respectively [13,14,15,16]. When the particles sinter and coalesce during the joining process, a dense joint seam consisting of bulk material is formed. Now the material retrieve its initial properties such as a high melting temperature [17,18]. Many examinations on nanojoining have been conducted with Ag and Cu nanoparticles in the field of electronic packaging [18,19,20,21], but this present investigation was focused on utilizing nanojoining in applications of structural loaded components. Therefore, Ni nanoparticles (abbreviated to “Ni-NP”) were used and processed into a high-metal-content suspension with respect to an easy handling and stabilization [22,23] of the particles before the actual joining process took place. These so-called nanopastes were applied on different base materials and then samples were joined. After shear strength testing, a great influence of the joining temperature on the results was revealed, but the joining pressure also affected the strength. The holding time on the other hand showed a minor influence and, in some cases, even an unexpected behavior, as the joining strength decreased at longer holding times for some base material/paste combinations. It was also found that the achieved strengths differed depending on the base material and the nanopaste used. 

## 2. Materials and Methods

### 2.1. Nanoparticles, Organics for Paste Preparation and Base Materials for Joining

**Figure 1:**
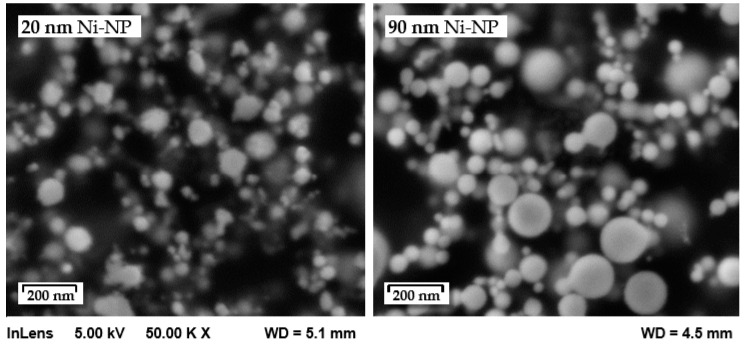
As-delivered nickel nanoparticles used for the preparation of Ni nanopastes.

Two different mean diameters of Ni-NP were used for this investigation, purchased from M K Impex Corp.(Mississauga, ON, Canada). According to the manufacturer, the mean particle sizes were 20 nm and 90 nm, which essentially could be verified by SEM images (Figure 1). 

Organics for the preparation of the Ni nanopastes, i.e., solvents and stabilizers, were screened in previous tests for volatility, viscosity, thermal behavior, etc. [11]. Based on the most promising organics from these investigations (Table 1), three different pastes were produced which were used in the present studies. The detailed compositions of the three nanopastes are listed in Table 2. Two of the pastes were based on PEG 400, which acted as solvent and stabilizer, while only varying the Ni-NP size. The third paste was a mixture of terpineol (solvent), KD4 (stabilizer) and 90 nm Ni-NP.

As base materials for the joining samples, three different nickel-base superalloys were used. The first one was Alloy 247 DS, a directionally solidified superalloy with a high γ’ (Ni_3_ [Al, Ti]) volume fraction. Furthermore, two versions of the widespread Inconel 718 were employed: one as a common cast material, which is referred to here as IN 718_c and another, additively manufactured, referred to here as IN 718_am.

### 2.2. Preparation of Ni Nanopastes

To produce nanopastes, the ingredients described in Table 2 were carefully weighed by placing them one after the other in a sample bottle. The metal content of 70 wt.% was chosen based on preliminary tests, as this content was determined to be the upper limit for reliable paste preparation for the 20 nm Ni-NP [26]. Although the content could be set higher for the 90 nm Ni-NP, the metal content of 70 wt.% was applied to all pastes to ensure the comparability of results. All ingredients in the bottle were then directly contacted to a sonotrode, which applied pulsed ultrasound of 24 kHz with one minute duration for an intensive dispersion (device: USP 200S, Hielscher-Ultraschall-Technologie, Teltow, GER). This was repeated five times whereby the paste was stirred manually between each cycle. The temperature was controlled at about room temperature by using a water bath around the bottle. The total mass of one batch produced this way was 2.5 g, several batches for every paste were prepared for whole investigations.

### 2.3. Manufacturing of Shear Test and Microstructure Samples as Well as Nanopaste Application

**Figure 2:**
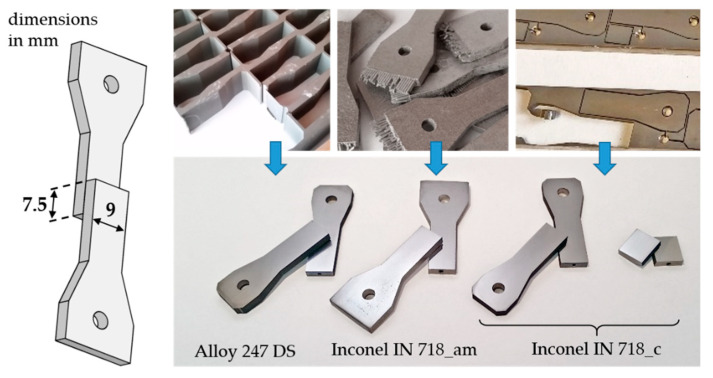
Sample geometry and base materials. Thickness of all samples: 3 mm.

First, the three different base materials were machined into parts with a 9 mm wide end on one side. After the actual joining process, two of these parts resulted in a shear test sample with an overlap length of 7.5 mm (joining area 67.5 mm^2^), see Figure 2. In order to use the material efficiently, IN 718_c was also cut into simple flat shaped pieces for metallographic investigations. IN 718_am was already delivered in a near net shape geometry.

**Figure 3:**
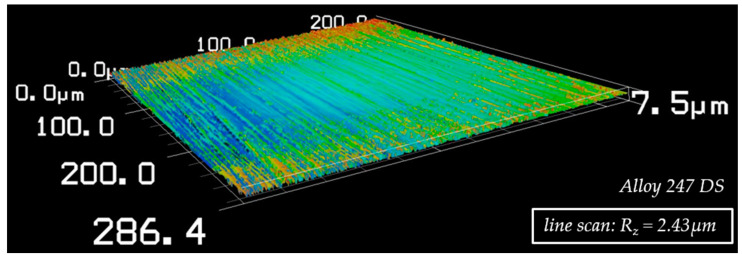
Microscopic structure of the finished sample surface (here Alloy 247 DS), captured via laser scanning microscope.

To create equal conditions for joining, the surfaces of all samples were finished by wet grinding using a SiC disc with grit 600. This was carried out while keeping the parts in a fixed alignment to the rotating grinding disc. The roughness of the so finished base materials surfaces was R_Z_ = 1.77–3.08 µm, captured via a line scan by using a laser scanning microscope. Figure 3 shows an example of Alloy 247 DS. 

Subsequently, the nanopaste was applied to the cleaned (via ethanol rinsing) surface of each sample part. Therefore, a sufficient amount of paste was spread evenly with a spatula first, then the sample was dragged beneath a peel bar, which set the nanopaste layer to a height of 50 µm. It should be noted that this paste application on both sample sides with a total of 100 µm did not represent the thickness of the final joint, which was reduced to around 10 to 20 µm after joining. The applied paste was then predried by heating the sample parts on a hot plate to 200 °C for 90 s for the PEG-based pastes and 140 °C for 90 s for the terpineol-based pastes, respectively. Those parameters were detected as near optimum in preliminary tests [26].

### 2.4. Joining Process

**Figure 4:**
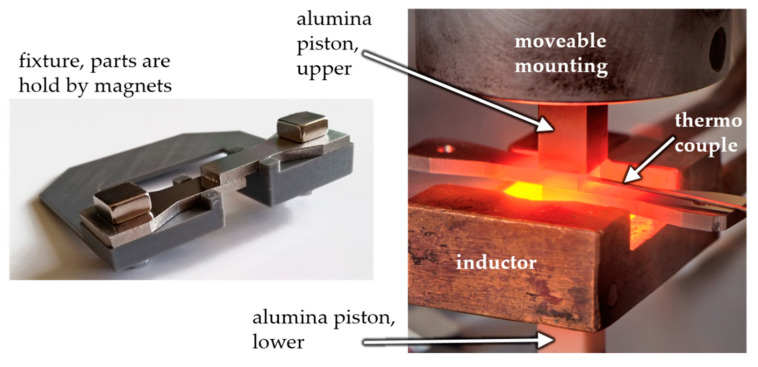
Sample part fixture (**left** side) and heated sample during joining process (**right** side).

Two nanopaste-coated sample parts were placed in a special fixture which can be seen in Figure 4 (left side). The so-positioned shear test sample was then introduced into the actual joining device in which the sample was clamped between a pair of alumina pistons before the fixture was removed. Joining was performed in a chamber that operated under a high vacuum at approx. 4 × 10^−4^ mbar or lower to avoid any impairment by air. At the same time, by means of a moveable upper piston, a joining pressure could be applied from the outside directly to the sample overlapping section. A thermocouple was fixed in a hole in the specimen, which was used to control temperature and time during the joining process. Heating took place inductively with a rate of 150 K/min, followed by free cooling after holding time. The section of the joining device where the sample was inserted is shown in Figure 4 (right side).

### 2.5. Joining Parameters DOE 

Since nanojoining, like brazing and similar processes, is based on the thermal effects of materials, the temperature of joining and holding time are crucial and, particularly in the case of nanojoining, the joining pressure too. Therefore, these three parameters were considered as the main parameters for the DOE. The range of investigation for those were set by preliminary tests. For example, it was found, that a joining temperature of approx. 675 °C represented the lower limit to obtain joined samples of at least handling strength (while holding time and joining pressure are at average values). Furthermore, the drop of the base material’s compression strength at high temperatures limited the maximum joining pressure [27]. In summary, the following ranges were covered for the investigation of shear strength: joining temperatures from 675 °C to 975 °C, holding time from 120 s (2 min) to 900 s (15 min) and joining pressure from 6.5 MPa to 40 MPa. For the holding time and joining pressure, one intermediate value (arithmetic mean) was added for the DOE. In the case of the joining temperature, the intermediate value was determined to be the typical heat-treatment temperatures of the respective materials for more sensible results. As a result, a temperature of 870 °C was selected for Alloy 247 DS and 760 °C for IN 718 (cast-type as well as additively manufactured). All of this is summarized in Table 3. 

### 2.6. Shear Strength Testing and Microstructure Analysis

The mentioned DOE was applied for all three base materials by using three different nanopastes, so 9 combinations in total. Each set of parameters was repeated on 2 to 3 samples for statistical validation. To shrink down the experimental expense, the DOE was statistically optimized. Nevertheless, around 400 samples were produced for shear testing and microstructure analysis. The shear samples were clamped in a testing machine (Zwick Allround-Line 20 kN) using a built-in vise which allowed a compensation of the vertical offset given by the overlapping geometry. The test was carried out at a quasi-static shear rate of 10^−3^/s (0.0075 mm/s). The initial force was 100 N for each sample, the test was then continued until the sample failed. 

The microstructure of the joints was analyzed by bright field images and differential interference contrast microscopy (DICM) using an Olympus GX51 (Hamburg, GER); SEM images were taken as well using Zeiss LEO 1455VP and Zeiss NEON 40EsB (Oberkochen, GER). In most cases, the topological structure was better recognized in DICM, so this was chosen over SEM for some images. 

## 3. Results and Discussion

### 3.1. Dependency of the Shear Strength on Base Material and Nanopaste

As an overview, the maximum shear strengths achieved at every base material/paste combination is shown in Table 4. Therefore, these results represent the highest value found in each of the nine sets of joint samples.

Among all samples made from Alloy 247 DS, a maximum shear strength of 154.5 Mpa was found. In contrast, joined samples from both materials IN 718_am and IN 718_c achieved up to 205.5 Mpa and 218.7 Mpa, respectively. To put this in perspective, by setting IN 718_c at 100%, IN 718_am corresponds to 94.0% and Alloy 247 DS to 70.6%. Thus, regardless of the nanopaste used, the joint samples of Alloy 247 DS showed the lowest values for shear strength. A reason could be the high volume fraction of the intermetallic phase γ’ (gamma prime), which is significantly higher for Alloy 247 DS with 62 vol.% [28] than for the Inconel materials with approx. 22 vol.% [29]. It is possible that the bonding of the seam to γ’, which must be considered as an intermetallic phase, is less strong than to the nickel solid solution of the base material. This would affect the joint strength. For brazing, the IN 718 is also known to exhibit better wettability and brazeability compared to Alloy 247 DS.

The three different nanopastes also affected the shear strengths of the joint samples significantly. By comparing the first two pastes Ni20_PEG and Ni90_PEG, the influence of the particle size was well indicated, since both differed only in the nanoparticle size used. For the paste Ni20_PEG (20 nm particles), the shear strengths across all base materials were lower than those for Ni90_PEG (90 nm particles). The difference was widest at base material Alloy 247 DS, where a maximum of only 53.0 MPa was achieved with paste Ni20_PEG and a maximum of 133.2 MPa with Ni90_PEG. This was similar for both Inconel 718 base materials, but the differences of shear strengths between the two pastes were smaller. This was unexpected, since from the theoretical behavior of nanoscale particles, smaller particles should sinter under less thermal energy (lower temperatures, shorter holding times) and thus lead to a bonding more easily than for larger ones. Therefore, better joining results were expected for the 20 nm Ni-NP. However, this was not confirmed in the course of this study and the opposite was the case. A possible explanation could be a different oxidation behavior of the particles. As for nickel in general, Ni-NPs formed an oxide shell in the presence of air. This could be more pronounced in the case of the 20 nm particles than for the larger ones, and also their specific surface area was larger, which further increased the oxide content. It can be assumed that this introduced more oxides into the joint seam, which hindered the sintering process of joining between the particles itself and to the base material. For definite statements, further research is needed.

The third paste also consisted of 90 nm Ni-NP, but here terpineol acted as a solvent and KD4 was used as a stabilizer. By employing this paste, higher shear strengths than for the PEG-based paste (also with 90 nm particles) could be achieved for all base materials. This showed that the chemical composition of the nanopastes also influenced the strength behavior, although to a lesser extent than the size of the nanoparticles used.

### 3.2. Dependency of the Shear Strength on Joining Parameters and Failure Behavior

This evaluation was carried out on six of the nine base material/paste combinations, since too many joint samples with paste Ni20_PEG (20 nm particles) turned out to fail immediately and therefore no suitable database was provided. Based on the available data, models were described for each combination of base material and paste in which the influence of joining parameters could be studied in detail using statistical software “Cornerstone 7.2”. Every model was formed on 12 measured shear strength values (each value based on the average of two or three repeated samples) as reference points. In order to avoid full spatial data plots, the dependencies found were broken down into categorized, non-numeric charts, see Table 5.

**Figure 5:**
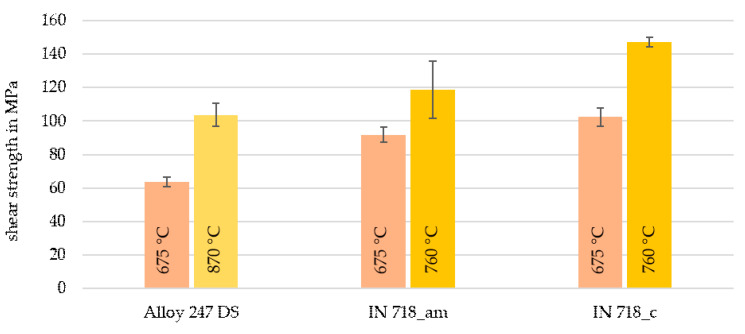
Shear strengths as a function of joining temperature (holding time: 15 min, joining pressure: 23.3 MPa) by using paste Ni90_T_KD4; the bar indications correspond to ± standard error.

The evaluation showed that a high joining temperature always led to a high shear strength, regardless of base material and paste. An approximately linear and strong increase in strength could be observed in the range examined (675–975 °C) for almost all combinations. In addition to shear strengths achieved at the highest joining temperature (given in Table 4), Figure 5 illustrates the influence of low and intermediate joining temperatures at a constant holding time and joining pressure of 900 s (15 min) and 23.3 MPa, respectively. In this comparison, the best performing paste Ni90_T_KD4 was used. The results show the enormous influence of the joining temperature.

This behavior also applied to the joining pressure for the most part, only the influence in some cases was not completely linear. In the case of IN 718_c + Ni90_T_KD4, high joining pressures resulted in a weaker and weaker increase of the shear strength, so that a plateau could ultimately be assumed. Basically, joining temperature and joining pressure behaved as expected in regard to nanojoining.

The holding time, which was also varied in the datasets as a main parameter, showed a weakly linear and also positive influence on the shear strength, but only in three of the six base material/paste combinations. In one case, a plateau formation could also be observed (Alloy 247 DS + Ni90_T_KD4). The other two combinations showed a more unusual behavior. For Alloy 247 DS + Ni90_PEG, a maximum shear strength occurred at intermediate values of holding time (approx. 9 min). For both shorter or longer holding times, the shear strength dropped. In the case of IN 718_am + Ni90_T_KD4, there was a tendency towards a negative influence, which meant that shorter holding times led to higher shear strengths, which indeed could be shown on individual parameter sets, see Table 6. In both, from set no. 085 to 087 and from 091 to 092, only the holding time was changed (increased), whereas the resulting average shear strengths in both cases showed a clear drop. Thus, this observation was not a random behavior, which required further investigations. Therefore, only an assumption can be given here. Since the joint seam consisted of almost pure nickel, perhaps a diffusion of the base material’s alloying elements into the seam took place. This could lead to a depletion of those elements at the near interface zone, which could further result in a weakening effect. However, it was not clear why this could not be observed for all combinations. Therefore, other explanations are of course also possible.

**Figure 6:**
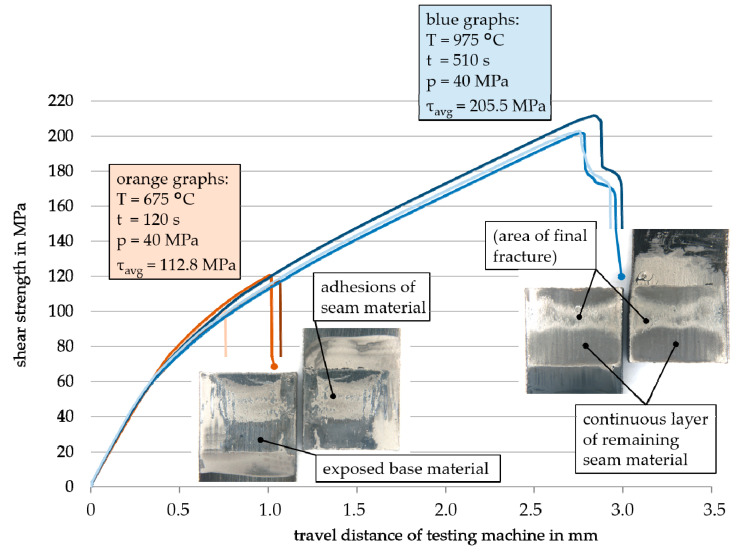
Strain curves for joint samples of two parameter sets (differentiated by orange and blue shades), in which the fracture surface of one sample is shown to indicate the different failure behaviors (base material: IN 718_am, nanopaste: Ni90_T_KD4).

In addition to the measurement of joining strengths, the strain curve was also recorded during the shear tests, which allowed further studies on their failure behavior. In addition, images were taken from the fractured surfaces, whereby two mechanisms could be identified. In the first one, shear samples with a low strength broke instantly after exceeding their maximum stress level. Although their fracture surface partially showed adhesions of joining seam material, the main area was equivalent to the exposed base material. Therefore, it can be assumed that the fracture took place through a detachment of the joint seam at the interface to the base material. The other mechanism was characterized by fracture surfaces of samples that achieved high strengths, on which almost no exposed base material could be seen. Instead, a continuous layer of remaining seam material adhered to both sides of the sample. Only a central part stood out which could be assigned to the area of final fracture. In these cases, the interface adhesion was no longer the weak point, and a higher force was transferred into the sintered joint seam itself until it broke. Accordingly, the corresponding strain curves showed a more ductile behavior, so that the fracture progressed gradually before the joint failed. These different failure behaviors are presented on the example of IN 718_am base material joined with Ni90_T_KD4 at different joining parameters in Figure 6. In this diagram, the strain curves for each of the three repeated samples of the two parameter sets are shown as well as both fracture surfaces of each sample.

### 3.3. Joint Microstructure

**Figure 7:**
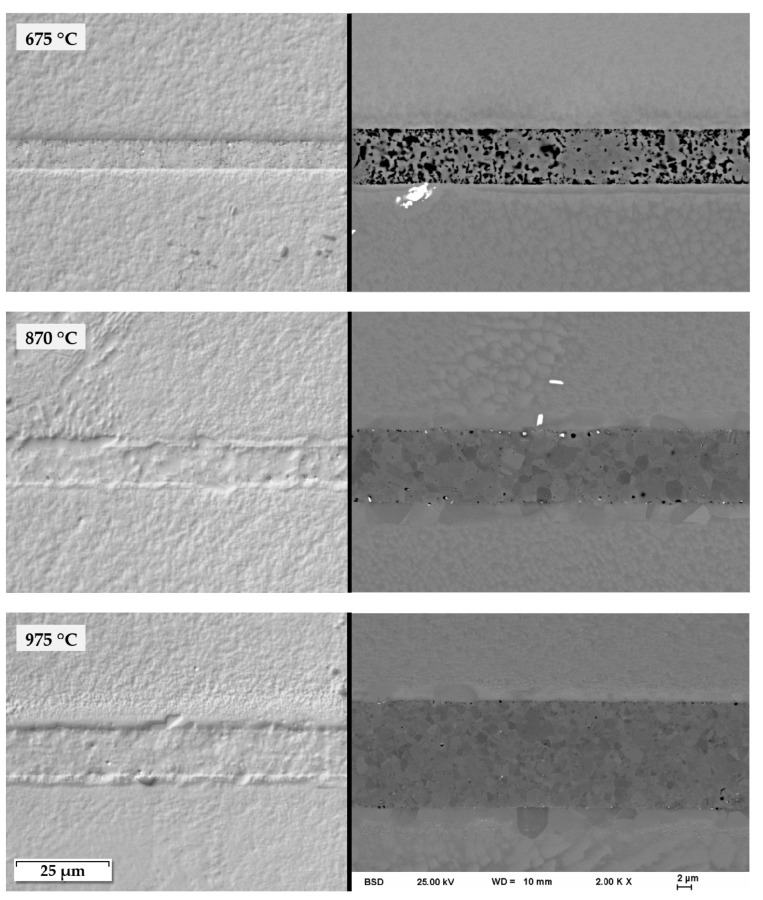
Microstructure of joining seams of Alloy 247 DS joined with Ni90_PEG at different temperatures with a holding time of 510 s (8.5 min) and a pressure of 23.3 MPa, light microscope (**left**) and SEM (**right**).

Depending on the joining parameters and base material/paste combination, the joints differed in porosity, defect density and adhesion to the base material. In general, low values for joining parameters resulted in a high porosity and poor adhesion. If those parameters were increased to a certain point, especially joining temperature, the microstructure of the joint seam became almost free of defects. For demonstration, in Figure 7, light microscopic DICM as well as SEM images were taken for the example of Alloy 247 DS + Ni90_PEG. The joining shown in this figure was carried out with a holding time of 510 s (8.5 min) and a joining pressure of 23.3 Mpa, whereas the joining temperature was varied between 675 °C, 870 °C and 975 °C. A clear change in the microstructure can be detected between joining temperatures of 675 °C and 870 °C. The density of the seam increases significantly. On the other hand, differences between the intermediate and higher temperature are smaller, but minor changes can be seen in the seam itself and at the interface zone. The formation of an additional layer at the interface at higher temperatures could be related to recrystallization effects. The thickness of the joint seam deviates a bit, depending on the point observed, and is not related to the various joint parameters.

**Figure 8:**
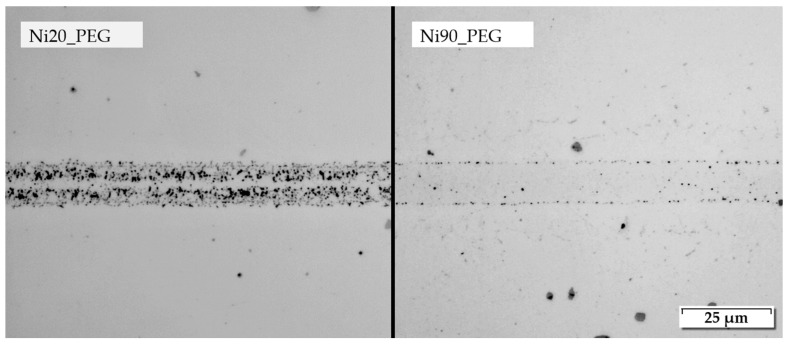
Microstructure of joining seams of IN 718_c using nanopastes Ni20_PEG and Ni90_PEG (joining temperature: 975 °C, holding time: 510 s (8.5 min), pressure: 23.3 MPa), light microscope.

It was also found that the two different Ni-NP average sizes of 20 nm and 90 nm led to a clearly different formation of joining seams. This could be seen in the comparison of the corresponding pastes Ni20_PEG and Ni90_PEG, which only differed in the Ni-NP size. In order to assess the light microscopy images, it is worth mentioning that not every dark spot within the seam was equal to a porosity or a void. Thus, for paste Ni20_PEG, significantly higher porosities or residues of the organics could be observed under the same joining conditions. Even when using optimal joining parameters, the structure of the seam was always characterized by more defects than for paste Ni90_PEG. The same applied to the bonding to the base material. Figure 8 shows a comparison of the two pastes Ni20_PEG and Ni90_PEG with base material IN 718_c joined at 975 °C (highest temperature) with a holding time of 510 s (8.5 min) and a joining pressure of 23.3 MPa. The observed microstructures also agree with the results of shear strength, where the use of paste Ni20_PEG led to lower values. Therefore, the assumption mentioned in Section 3.1 reflected that a stronger oxidation of the smaller particles led to an insufficient sintering of the particles and thus to lower strengths. Despite the microstructure exhibiting much more defects when using paste Ni20_PEG, a maximum shear strength of 131.3 MPa was still achieved for IN 718_c.

By using either paste Ni90_PEG or paste Ni90_T_KD4, the joint seams pointed to a high density and bonded to every base material even at only intermediate joining temperatures. Moreover, the formation of these seams could be observed even at the shortest holding time of 2 min. Between the two 90 nm Ni-NP pastes in general, no remarkable differences in the formation of the joint structure could be seen, although the maximum achievable shear strengths of samples using paste Ni90_T_KD4 were slightly higher. 

**Figure 9:**
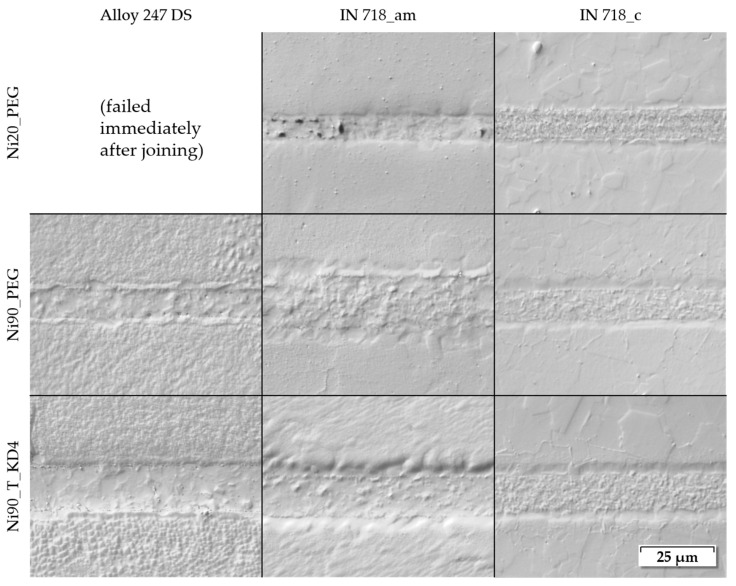
Microstructure of joining seams of all base materials/paste combinations (joining temperature: 870 °C, holding time: 510 s (8.5 min), pressure: 23.3 MPa), light microscope (DICM).

The following light microscopy (DICM) images (Figure 9) show the joint seam formation for all nine combinations of the three investigated nanopastes with all three base materials for a joining temperature of 870 °C. For this comparison, the holding time and joining pressure were also fixed at their intermediate values at 510 s (8.5 min) and 23.3 MPa, respectively. Some minor differences could be observed in the joint microstructure of the different base material/paste combinations. There were variations in the grain structure of the seam, but these also occurred along a single seam. Therefore, across all combinations, the seams were formed similarly, and significant differences could not be detected, apart from paste Ni20_PEG which, as already mentioned, led to more defects than the other pastes. In particular, there were no visual abnormalities regarding the lower shear strength that were found for samples made of Alloy 247 DS. However, for certain low joining parameters the adhesion between Alloy 247 DS and the joint seam was a little more frequently affected by defects in comparison to the other base materials, but for intermediate and higher parameter ranges there were no significant differences.

Further, the appearance of the additional layer at the interface between the base material and the seam, which was most likely caused by recrystallization effects, varied slightly depending on the base material due to their different properties.

## 4. Conclusions

In this study, the joining of three different nickel-base superalloys using different Ni nanopastes in a vacuum process was investigated in detail. Samples were examined for their shear strength and joint microstructure by varying the joining temperature, holding time and joining pressure.

Overall, the feasibility of joining nickel-base alloys with nanopastes at comparatively low temperatures could be shown. Even for the lowest examined joining temperature (675 °C), shear strengths of over 100 MPa were achieved, at least in the case of the two Inconel materials. The shear strengths increased significantly with higher values for the joining temperature and pressure, whereas the holding time partially showed an unusual behavior, which was described in more detail. The maximum shear strength determined within this investigation was 218.7 MPa for the base material IN 718_c joined with paste Ni90_T_KD4. In addition, it was found that the nanopastes with 90 nm particles unexpectedly led to better results than the paste with 20 nm particles. Possible reasons for this were discussed. Moreover, the chemical composition of the nanopastes also influenced the strengths.

Two tendencies could be identified for the failure behavior of the joining samples in the shear tests. Low-shear-strength samples broke instantly mainly by detachment between the joint seam and base material, which was indicated by the exposed base material on the fracture surface. For samples with a higher shear strength, the strain curve showed a rather ductile failure behavior with gradual crack propagation. In these cases, the sample’s fracture surfaces were evenly covered with the remaining adhesions of the seam, which suggested a fracture within the sintered joint.

The microstructure of the joints appeared to be almost defect-free if appropriately joining parameters were used. However, e.g., for low temperatures or in the case of the paste with 20 nm particles, the porosity and adhesion defect density were increased. For the two pastes based on 90 nm Ni-NP, seams showed a high density even at only intermediate joining temperatures and a short holding time of 2 min. When varying the base materials, hardly any differences in the joint microstructure could be detected.

Further steps are planned to investigate how the bonding to the base material can be improved, since this represents a weak point, at least at low joining temperatures. Fluoride ion cleaning (FIC) would be conceivable here. In order to facilitate the applicability of the process in practice, the possibilities of reducing the joining pressure while keeping the joint properties at a sufficient level should be studied.

## Figures and Tables

**Table 1:** Organics used for preparation of Ni nanopastes.

Compound	Formula	CAS No.	Supplier	Usage and Ref.
Polyethylene glycol (PEG) 400	C_2n_H_4n+2_O_n+1_	25322-68-3	Carl Roth GmbH (Germany)	Solvent and stabilizer [24]
Terpineol pure	C_10_H_18_O	8000-41-7	Carl Roth GmbH (Germany)	Solvent [25]
Hypermer™ KD4	-	-	Croda Int. Plc (UK)	Stabilizer [25]

**Table 2:** Composition of the nanopastes.

Description of Nanopaste	Metal Content	Organics
Ni20_PEG	20 nm Ni-NP, 70 wt.%	PEG 400, bal.
Ni90_PEG	90 nm Ni-NP, 70 wt.%	PEG 400, bal.
Ni90_T_KD4	90 nm Ni-NP, 70 wt.%	Solvent: terpineol pure, bal. Stabilizer: Hypermer™ KD4, 0.7 wt.%

**Table 3:** Investigated parameter ranges for joining samples.

Base Material	Joining Temperatures (°C)	Holding Times (s)	Joining Pressures (MPa)
**Alloy 247 DS**	675/870/975	120/510/900	6.5/23.3/40
**IN 718_am** and **IN 718_c**	675/760/975		

**Table 4:** Achieved maximum shear strengths by all base material/paste combinations. Value sets below the shear strength represent the corresponding joining parameters: T (°C)/t (s)/p (MPa).

Joined with Nanopaste	Alloy 247 DS	IN 718_am (Additively Manufactured)	IN 718_c (Common)
**Ni20_PEG**	53.0 MPa (975/900/6.5)	155.1 MPa (975/510/40)	131.3 MPa (975/120/23.3)
**Ni90_PEG**	133.2 MPa (975/510/40)	181.8 MPa (975/510/40)	181.2 MPa (975/510/40)
**Ni90_T_KD4**	154.5 MPa (975/510/40)	205.5 MPa (975/510/40)	218.7 MPa ^1^ (975/510/40)

^1^ Represents the highest value of shear strength of the whole investigation.

**Table 5:** Influence of joining parameters on shear strength as non-numerical charts (T: joining temperature, t: holding time, p: joining pressure).

Joined with Nanopaste	Alloy 247 DS	IN 718_am (Additively Manufactured)	IN 718_c (Common)
**Ni90_PEG**	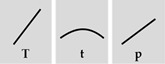	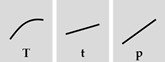	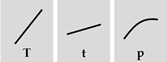
**Ni90_T_KD4**	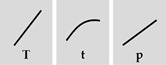	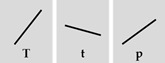	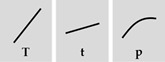

**Table 6:** Results of base material/paste combination IN 718_am + Ni90_T_KD4 with significant value changes highlighted (bold).

Parameter Set				Achieved Shear Strength			
	Joining Temp.	Holding Time	Joining Pressure	Sample #1	Sample #2	Sample #3	Avg. #1–3
No.	(°C)	(s)	(MPa)	(MPa)	(MPa)	(MPa)	(MPa)
FV_085	760	**120**	6.5	78.6	96.7	119.6	**98.3**
FV_087	760	**510**	6.5	60.8	79.0	70.6	**70.1**
FV_091	675	**510**	23.3	113.9	82.0	122.1	**106.0**
FV_092	675	**900**	23.3	80.7	96.9	97.6	**91.7**
